# Electrocardiography Score for Left Ventricular Systolic Dysfunction in Non-ST Segment Elevation Acute Coronary Syndrome

**DOI:** 10.3389/fcvm.2021.764575

**Published:** 2022-01-07

**Authors:** Wei-Chen Lin, Ming-Chon Hsiung, Wei-Hsian Yin, Tien-Ping Tsao, Wei-Tsung Lai, Kuan-Chih Huang

**Affiliations:** ^1^Heart Center, Cheng Hsin General Hospital, Taipei, Taiwan; ^2^Department of Internal Medicine, Keelung Hospital, Ministry of Health and Welfare, Keelung, Taiwan; ^3^Faculty of Medicine, School of Medicine, National Yang Ming Chiao Tung University, Taipei, Taiwan; ^4^Division of Cardiology, Department of Internal Medicine, National Defense Medical Center, Tri-Service General Hospital, Taipei, Taiwan; ^5^Graduate Institute of Clinical Medicine, College of Medicine, National Taiwan University, Taipei, Taiwan; ^6^Section of Cardiology, Department of Internal Medicine, National Taiwan University Hospital Hsin-Chu Branch, Hsin-Chu, Taiwan; ^7^Department of Internal Medicine, National Taiwan University Hospital, Taipei, Taiwan

**Keywords:** electrocardiography, left ventricular systolic dysfunction, NSTE-ACS, cardiac point of care ultrasounds, GRACE, TIMI

## Abstract

**Background:** Few studies have characterized electrocardiography (ECG) patterns correlated with left ventricular (LV) systolic dysfunction in patients with non-ST segment elevation acute coronary syndrome (NSTE-ACS).

**Objectives:** This study aims to develop ECG pattern-derived scores to predict LV systolic dysfunction in NSTE-ACS patients.

**Methods:** A total of 466 patients with NSTE-ACS were retrospectively enrolled. LV ejection fraction (LVEF) was assessed by echocardiography within 72 h after the first triage ECG acquisition; there was no coronary intervention in between. ECG score was developed to predict LVEF < 40%. Performance of LVEF, the Global Registry of Acute Coronary Events (GRACE), Thrombolysis in Myocardial Infarction (TIMI) and ECG scores to predict 24-month all-cause mortality were analyzed. Subgroups with varying LVEF, GRACE and TIMI scores were stratified by ECG score to identify patients at high risk of mortality.

**Results:** LVEF < 40% was present in 20% of patients. We developed the PQRST score by multivariate logistic regression, including **p**oor R wave progression, **Q**RS duration > 110 ms, heart **r**ate > 100 beats per min, and **ST-**segment depression ≥ 1 mm in ≥ 2 contiguous leads, ranging from 0 to 6.5. The score had an area under the curve (AUC) of 0.824 in the derivation cohort and 0.899 in the validation cohort for discriminating LVEF < 40%. A PQRST score ≥ 3 could stratify high-risk patients with LVEF ≥ 40%, GRACE score > 140, or TIMI score ≥ 3 regarding 24-month all-cause mortality.

**Conclusions:** The PQRST score could predict LVEF < 40% in NSTE-ACS patients and identify patients at high risk of mortality in the subgroups of patients with LVEF ≥ 40%, GRACE score > 140 or TIMI score ≥ 3.

## Introduction

Non-ST segment elevation acute coronary syndrome (NSTE-ACS) includes unstable angina and non-ST segment elevation myocardial infarction (NSTEMI). Its incidence increases continuously. Moreover, NSTEMI accounts for more than half of the acute myocardial infarction proportion, becoming a significant burden to public health (1, 2). Left ventricular ejection fraction (LVEF) was not included in previous models for risk stratification in patients with NSTE-ACS (3, 4). However, its reduction portends a worse prognosis independent of clinical heart failure (5–7). Recently, the identification of left ventricular (LV) systolic dysfunction has been emphasized in practice guidelines, and LVEF < 40% should trigger an early invasive strategy in NSTE-ACS patients (8).

The timing of echocardiographic examination was also emphasized in recent evidence-based studies of acute myocardial infarction (6, 9). Previous registry studies revealed that more than 30% of NSTE-ACS patients did not undergo echocardiographic examination at the index events, and the timing of those performed echocardiograms was rarely reported (10). Cardiac point-of-care ultrasound (POCUS) provides immediate detection of LV systolic dysfunction and structural information and avoids unstable patient transportation, especially in the coronavirus disease-2019 (COVID-19) pandemic era (11, 12). However, the steep learning curve and technical pitfalls prevent cardiac POCUS democratization in NSTE-ACS settings (13, 14).

Electrocardiogram (ECG) is a standard diagnostic tool for acute chest pain with high availability and instantaneity. Most physicians can recognize important abnormal ECG markers representing conduction abnormalities and myocardial ischemia. Previous studies demonstrated that abnormal ECG patterns predicted left ventricular systolic dysfunction in various populations (15–17); thus, we hypothesize that an ECG pattern-derived score that predicts LV systolic dysfunction should make cardiac POCUS a more efficient risk stratification tool in NSTE-ACS.

The study aims to develop an ECG pattern score to predict LV systolic dysfunction in NSTE-ACS and investigate its additional prognostic value.

## Materials and Methods

### Study Design and Population

Patients admitted consecutively for NSTE-ACS at Cheng Hsin General Hospital from January 2018 to April 2020 were retrospectively enrolled in the overall cohort. Those from January 2018 to October 2019 were included in the derivation cohort, and those from November 2019 to April 2020 were included in the validation cohort. The Institutional Review Board at Cheng Hsin General Hospital approved the study. All NSTE-ACS events were diagnosed according to the 2020 ESC guidelines (18). Exclusion criteria included out-of-hospital cardiac arrest, postheart transplantation status, pacemaker rhythm, missing ECG at the emergency room triage, and poor echocardiographic quality. Baseline demographic and clinical characteristics and laboratory data of the study subjects were obtained from electronic medical records. Because LV function can alternate by either coronary revascularization or conservative treatments, the interval between the triage ECG tracings and echocardiograms for individual patients in this study was all within 72 h; there were no coronary interventions between.

### ECG Analysis

All standard 12-lead ECG tracings at index hospitalization for NSTE-ACS were obtained before echocardiography. The 12-lead ECGs were calibrated at 1 mm = 0.1 mV and recorded with a paper speed of 25 mm/s. Nine ECG candidate patterns were analyzed to predict LVEF < 40%: Q waves in 2 or more contiguous leads (Q-wave with a duration ≥ 40 ms and/or a depth ≥ 25% of the R-wave in the same lead) (17), atrial fibrillation (17), ST-segment depression (STD) ≥ 0.1 mV in at least 2 contiguous leads (19) (ST-segment deviation was measured at 60–80 ms after the J point in all leads except aVR), poor R wave progression (defined as R wave amplitude ≤ 0.3 mV in lead V3 and R wave amplitude in lead V2 equal or less than the R wave amplitude in lead V3) (20), low voltage (defined as the amplitudes of all ORS complexes in limb leads < 0.5 mV or the amplitudes of all ORS complexes in precordial leads < 1.0 mV) (21), heart rate (HR) more than 100 beats per minute (bpm), QRS duration > 110 ms (15) [both left bundle branch block (22) and right bundle branch block (23) were also included], left ventricular hypertrophy according to the Sokolow-Lyon criteria or Cornell criteria (24, 25) and left atrial enlargement (LAE) (26) (defined as notched P wave with interpeak duration > 40 ms on lead II or area of negative P wave in lead V1 > 40 ms x 1 mm) (27). Two experienced cardiologists confirmed all patterns.

### Echocardiography

Transthoracic echocardiograms (TTE) at index hospitalization from January 2018 to April 2020 were retrospectively investigated via PACS (Intellispace Cardiovascular, Philips Medical Systems, Andover, MA). Cardiologists performed all cardiac POCUS with a Philip CX50 machine and S5-1 transducer. Patients with LVEF < 40% by the biplane method of disk summation were considered to have reduced LV systolic function.

### Statistical Analysis

#### Predictive Model Development

Categorical variables are presented as frequencies and proportions and were compared using χ2 tests. Continuous variables are described as the mean ± standard deviation (SD) and were compared using Student's *t-*test. We evaluated the association of nine ECG patterns with LVEF < 40% to develop a predictive score model. The candidate patterns with *p* < 0.10 in the χ2 test were included in multivariate logistic regression. Regression coefficients and odds ratios (ORs) were described with 95% confidence intervals (CIs). The regression coefficients of the candidate variables with *p* < 0.05 in multivariate logistic regression were used to assign the predictive score points. Each weight of the variable was taken as the corresponding regression coefficient divided by the smallest coefficient and rounded to the nearest integer or half-integer. The sum of the weight of each variable gave an individual risk estimate. The prediction score's discriminative power was tested by calculating the area under the receiver operating characteristic (ROC) curves (AUCs). The scoring model was internally validated. A separate small cohort was used to externally validate the score. The cut-off value was obtained with its specificity at least more than 90% and the maximal corresponding sensitivity. Positive predictive value (PPV) and negative predictive value (NPV) were calculated based on standard methods.

#### Survival Analysis of LVEF, ECG, GRACE, and TIMI Scores

To test the prognostic value of the ECG score, we referred to LVEF, GRACE and TIMI scores as comparative predictors. The performance of each predictor for the discrimination of 24-month all-cause mortality was tested by ROC curves. The comparison between AUCs of predictors was performed by DeLong test. The 24-month survival estimates of all-cause mortality were described with the Kaplan-Meier method and stratified by LVEF ≥ 40% and < 40%, GRACE score > 140 and GRACE ≤ 140 (28), or TIMI score ≥ 3 and TIMI < 3 (29). The survival analysis stratified by the aforementioned ECG score was also described with a determined cutoff value. Cumulative event rates were compared by log-rank test. If a significant difference in survival estimates was attained by the ECG score, predictive value of the ECG score for 24-month all-cause mortality was analyzed by multivariate Cox regression in subgroups based on varying LVEF (LVEF < 40 and ≥ 40%), GRACE score (GRACE > 140 and ≤ 140), and TIMI score (TIMI ≥ 3 and TIMI < 3). Interaction test was performed to confirm the differences between subgroups. The results were presented as hazard ratio (HR) and 95% CI. All analyses were considered statistically significant if the *P* value was < 0.05. Data were analyzed using IBM SPSS statistical software for Macintosh, version 25 (Armonk, NY: IBM Corp.) and MedCalc Statistical Software version 20.015 (MedCalc Software bv, Ostend, Belgium).

## Results

### Patient Characteristics

The demographic and clinical features of patients with LVEF ≥ 40% and LVEF < 40% in our cohort was provided in Table 1. There were 466 patients enrolled in the overall cohort. The incidence of LVEF < 40% was 20.4% (95/466). Patients with LVEF < 40% were older and had more comorbidities, including diabetes mellitus, old cerebrovascular accidents, and chronic kidney disease (eGFR < 60 ml/min/1.73m^2^). Patients with LVEF < 40% were more prone to triple-vessel coronary artery disease at the index coronary angiography, a higher CABG rate, and a longer hospital stay. A total of 58.8% (274/466) of patients accepted cardiac POCUS at the emergency room or coronary care unit. Three Hundred and fifty three patients admitted between January 2018 and October 2019 were included in the derivation cohort, and 113 patients admitted between November 2019 and April 2020 were included in the validation cohort. Their baseline characteristics were described in Table 2.

**Table 1:** Baseline characteristics in the overall cohort.

	**LVEF ≥ 40%**	**LVEF <40%**	***P*-value**
	**(*n* = 371)**	**(*n* = 95)**	
Age, years	64.5 ± 11.7	71.3 ± 11.7	<0.001
Male, *n* (%)	297 (80.1)	76 (80)	0.991
Hypertension, *n* (%)	240 (64.7)	63 (66.3)	0.767
Dyslipidemia, *n* (%)	213 (57.4)	51 (53.7)	0.513
Diabetes, *n* (%)	147 (39.6)	52 (54.7)	0.008
Old CVA, *n* (%)	17 (4.6)	10 (10.5)	0.027
CKD, *n* (%)	86 (23.2)	46 (48.4)	<0.001
Prior PCI, *n* (%)	72 (19.4)	18 (18.9)	0.919
Prior CABG, *n* (%)	31 (8.4)	14 (14.7)	0.060
CAG, *n* (%)	350 (94.3)	87 (91.6)	0.320
PCI, *n* (%)	266 (71.7)	49 (51.6)	<0.001
CABG, *n* (%)	47 (12.7)	33 (34.7)	<0.001
Triple vessel disease at index CAG, *n* (%)^*^	224 (64)	73 (83.9)	<0.001
Total cholesterol, mg/dL	179 ± 72.5	171.9 ± 40.9	0.362
Triglyceride, mg/dL	167.1 ± 147.8	132 ± 77.7	0.002
HDL-C, mg/dL	39.4 ± 14.2	39.5 ± 11.2	0.933
LDL-C, mg/dL	110.2 ± 43.2	106.6 ± 36.7	0.461
**Predischarge drug administration**			
Antiplatelet, *n* (%)^†^	360 (98.4)	89 (100)	0.224
ACEI/ARB, *n* (%)^†^	222 (60.7)	59 (66.3)	0.326
Beta-blocker, *n* (%)^†^	236 (64.5)	69 (77.5)	0.019
Statin, *n* (%)^†^	328 (89.6)	64 (71.9)	<0.001
TIMI score	3.7 ± 1.4	4.4 ± 1.5	<0.001
GRACE score	127.7 ± 35.1	166 ± 38.6	<0.001
Hospital stays, days	7.1 ± 8.5	14 ± 15.9	<0.001
Peak troponin I, ng/mL	21.5 ± 98.1	22.2 ± 41.7	0.948

*n, number; SD, standard deviation; old CVA, old cerebrovascular accident; CKD, chronic kidney disease, defined as eGFR < 60 ml/min/1.73 m^2^; PCI, percutaneous coronary intervention; CABG, coronary artery bypass graft; CAG, coronary angiography; HDL-C, high density lipoprotein-Cholesterol; LDL-C, low density lipoprotein-Cholesterol; LVEF, left ventricular ejection fraction; ACEI, angiotensin converting enzyme inhibitor; ARB, angiotensin II receptor blockers; TIMI, Thrombolysis In Myocardial Infarction; GRACE, The Global Registry of Acute Coronary Events*.

* *n (denominator) = 350 for the group with LVEF ≥ 40% and n = 87 for the group with LVEF < 40%*.

† *n = 366 for the group with LVEF ≥ 40% and n = 89 for the group with LVEF < 40%. (11 patients expired at index hospitalization)*.

**Table 2:** Baseline characteristics in the derivation and validation cohorts.

	**Derivation cohort**	**Validation cohort**	***P*-value**
	**(*n* = 353)**	**(*n* = 113)**	
Age, years	66.1 ± 12.1	65.4 ± 11.8	0.617
Male, *n* (%)	280 (79.3)	93 (82.3)	0.490
Hypertension, *n* (%)	227 (64.3)	76 (67.2)	0.567
Dyslipidemia, *n* (%)	198 (56.1)	66 (58.4)	0.665
Diabetes, *n* (%)	144 (40.8)	55 (48.7)	0.141
Old CVA, *n* (%)	19 (5.4)	8 (7)	0.502
CKD, *n* (%)	95 (26.9)	37 (32.7)	0.231
Prior PCI, *n* (%)	74 (21)	16 (14.2)	0.111
Prior CABG, *n* (%)	34 (6.6)	11 (9.7)	0.974
CAG, *n* (%)	334 (94.6)	103 (91.2)	0.184
PCI, *n* (%)	238 (67.4)	77 (68.l)	0.887
CABG, *n* (%)	63 (17.8)	17 (15)	0.492
Triple vessel disease at index CAG, *n* (%)^*^	224 (67.1)	73 (70.9)	0.469
LVEF, %	51.0 ± 12.4	51.9 ± 13.7	0.538
LVEF <40%, *n* (%)	67 (19)	28 (24.8)	0.183
RWMA, *n* (%)	189 (53.5)	53 (46.9)	0.219
Total cholesterol, mg/dL	178.1 ± 73.1	176 ± 45.2	0.769
Triglyceride, mg/dL	160.4 ± 143.2	159.1 ± 117.6	0.930
HDL-C, mg/dL	39.6 ± 14.3	39 ± 11.4	0.720
LDL-C, mg/dL	109.4 ± 43	109.6 ± 38.4	0.960
TIMI score	3.8 ± 1.4	4.0 ± 1.6	0.189
GRACE score	136 ± 38.4	134.4 ± 41.0	0.708
Hospital stays, days	8.5 ± 10.1	8.7 ± 12.7	0.833
Peak troponin I, ng/mL	21.8 ± 100.7	21.4 ± 36.2	0.971

*RWMA, regional wall motion abnormalities. Other abbreviations as in Table 1*.

* *n (denominator) = 334 in the derivation cohort and n = 103 in the validation cohort*.

### ECG Analysis in the Derivation Cohort

The associations between LVEF < 40% and nine ECG patterns were investigated by the chi-square test (Table 3). Seven patterns with a *p*-value < 0.1 (Q waves in 2 or more contiguous leads, atrial fibrillation, STD ≥ 0.1 mV in 2 or more contiguous leads, PRWP, QRS duration > 110 ms, HR > 100 bpm, and LAE) were considered candidate predictors and adopted for multivariate logistic regression to predict LVEF < 40%. Four of these 7 predictors independently remained significant predictors: PRWP (OR 5.550, CI 2.671–11.531), QRS duration > 110 ms (OR 4.115, CI 1.814–9.332), HR > 100 bpm (OR 4.030, 1.970–8.245), and STD ≥ 1 mm in ≥ 2 contiguous leads (OR 2.230, CI 1.175–4.234) (Table 4).

**Table 3:** Distribution of ECG variables and chi-square analysis according to LVEF in the derivation cohort.

**ECG variables**		**LVEF ≥ 40%**	**LVEF <40%**	***P*-value**
		**(*n* = 286)**	**(*n* = 67)**	
Q wave ≥ 2 contiguous leads	yes	72	25	0.045
	no	214	42	
Atrial fibrillation	yes	10	8	0.005
	no	276	59	
STD ≥ 1 mm in ≥ 2 contiguous leads	yes	79	35	<0.001
	no	207	32	
PRWP	yes	53	35	<0.001
	no	233	32	
Low voltage	yes	12	6	0.111
	no	274	61	
HR > 100 bpm	yes	31	28	<0.001
	no	255	39	
QRS duration > 110 ms^*^	yes	22	23	<0.001
	no	264	44	
LVH	yes	33	11	0.276
	no	253	56	
LAE	yes	9	7	0.010
	no	277	60	

*ECG, electrocardiography; LVEF, left ventricular ejection fraction; STD, ST-segment depression; PRWP, poor R wave progression; HR, heart rate; LVH, left ventricular hypertrophy; LAE, left atrial enlargement*.

* *QRS duration > 110 ms included 24 complete right bundle branch blocks (53.3%), 7 complete left bundle branch blocks (15.6%) and 14 non-specific intraventricular conduction delays (31.1%)*.

**Table 4:** Multivariate logistic regression analysis predicting LVEF < 40% in the derivation cohort and point assignment based on the regression coefficient.

**ECG variables**	**Beta coefficient**	***P*-value**	**OR (95% CI)**	**Point assigned**
Q wave ≥ 2 contiguous leads	−0.113	0.770	0.893 (0.419–1.905)	
Atrial fibrillation	0.072	0.916	1.074 (0.283–4.074)	
STD ≥ 1 mm in ≥ 2 contiguous leads	0.802	0.014	2.230 (1.175–4.234)	1
PRWP	1.714	<0.001	5.550 (2.671–11.531)	2
HR > 100 bpm	1.394	<0.001	4.030 (1.970–8.245)	1.5
QRS duration > 110 ms	1.415	0.001	4.115 (1.814–9.332)	2
LAE	0.050	0.939	1.051 (0.291–3.799)	

*Abbreviations as in Table 3*.

### The PQRST Score to Predict LVEF < 40%

**Figure 1:**
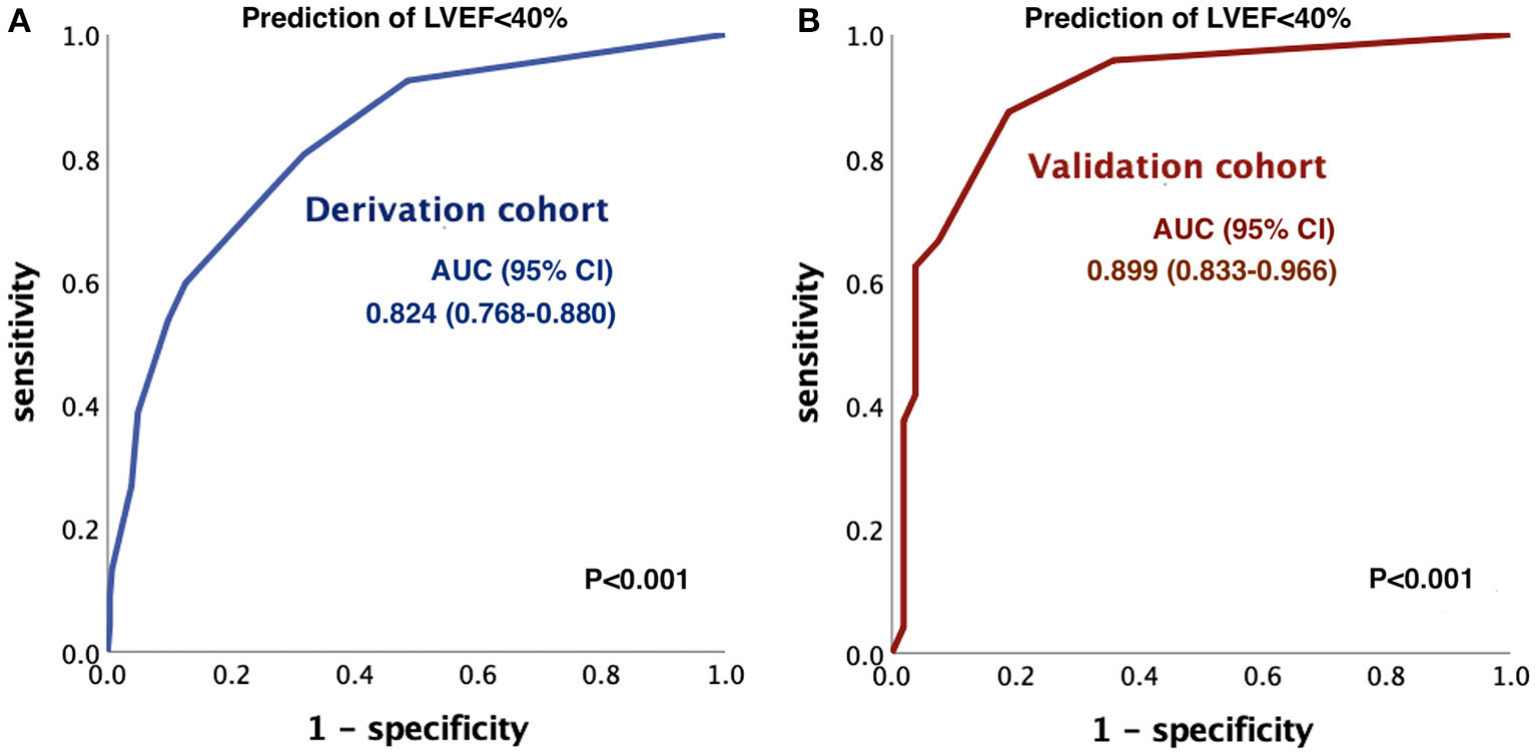
ROC curve analysis of the PQRST score to predict LVEF < 40%. The AUC was **(A)** 0.824 (95% CI 0.768–0.880) in the derivation cohort and **(B)** 0.899 (95% CI 0.833–0.966) in the validation cohort. ROC, receiver operating characteristic; AUC, area under the curve; CI, confidence interval; LVEF, left ventricular ejection fraction.

The score was denoted as the PQRST score (PWRP, QRS, heart rate, ST-segment depression, Table 4). According to the results of multivariate logistic regression, 2 points were assigned for PRWP, 2 points for QRS duration > 110 ms, 1.5 points for heart rate > 100 bpm, and 1 point for STD ≥ 0.1 mV in ≥ 2 contiguous leads. The highest risk score will be 6.5 points. By ROC curve analysis, the AUC values of the PQRST score in predicting LVEF < 40% were 0.824 (95% confidence interval (CI): 0.768–0.880; cutoff 3 at sensitivity 53.7%, specificity 90.2%, PPV 56.3% and NPV 89.3%) in the derivation cohort and 0.899 (95% CI: 0.833–0.966) in the validation cohort (Figure 1). The proportion of LVEF < 40% in subjects with PQRST ≥ 3 was 56.3% (36/64) in the derivation cohort, and the proportion of LVEF < 40% in those with PQRST ≥ 3 was 75% (18/24) in the validation cohort.

### Performance of LVEF, PQRST, GRACE and TIMI Scores for Discrimination of 24-Month All-Cause Mortality by ROC Curve Analysis

**Figure 2:**
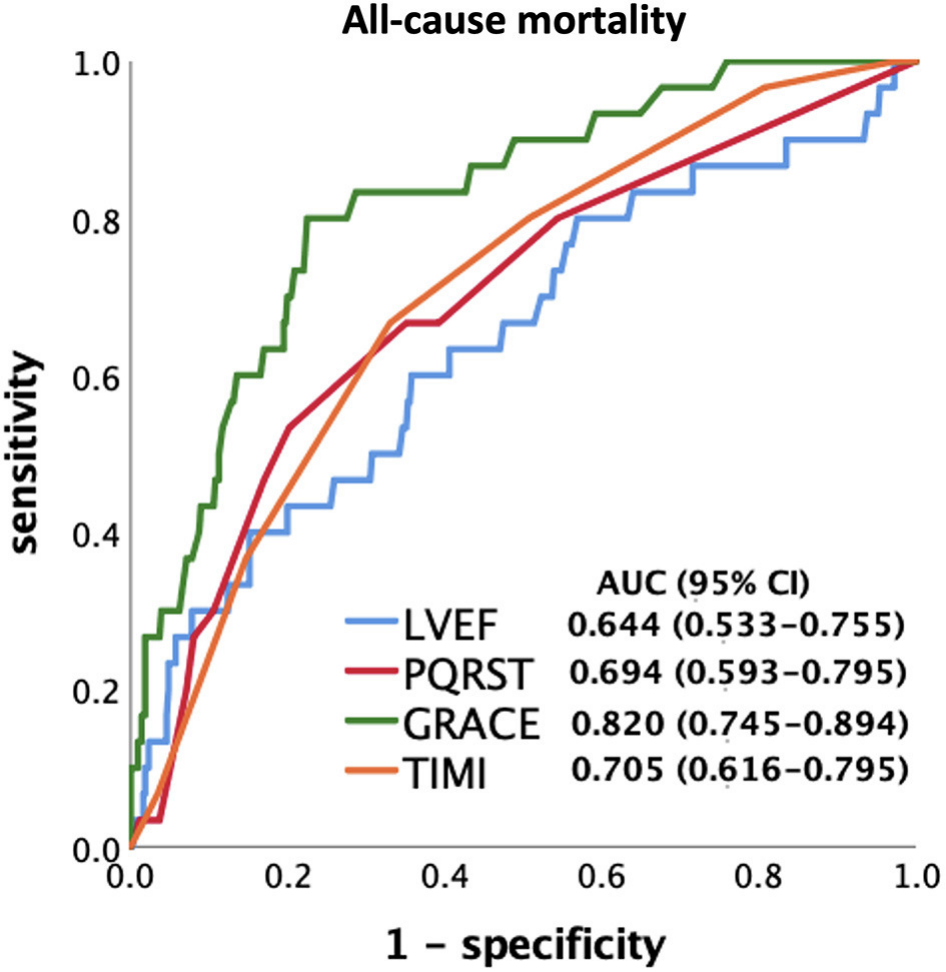
ROC curve analysis of LVEF, PQRST, and traditional predictive models to predict 24-month all-cause mortality. All predictors attained a significant *p*-value (*p* < 0.05), and the GRACE score had the highest AUC value in the study endpoints. TIMI, Thrombolysis In Myocardial Infarction; GRACE, The Global Registry of Acute Coronary Events.

ROC analysis was performed to discriminate all-cause mortality at 24 months (Figure 2). The AUC values were 0.644 for LVEF (95% CI: 0.533–0.755, *p* =0.008), 0.694 for PQRST (95% CI: 0.593–0.795, *p* < 0.001), 0.820 for GRACE (95% CI: 0.745–0.894, *p* < 0.001) and 0.705 for TIMI (95% CI: 0.616–0.795, *p* < 0.001). Comparison between AUCs of predictors was performed by Delong test, described in Table 5. The PQRST score seemed to have less predictive value for 24-month all-cause mortality than the GRACE score (difference between area: 0.126; 95% CI: 0.048–0.203, *p* = 0.002) but have similar value as TIMI score and LVEF (difference between area: 0.011; 95% CI:−0.106–0.129, *p* = 0.850, compared to TIMI score, and 0.050; 95% CI:−0.051–0.150, *p* = 0.331, compared to LVEF).

**Table 5:** Comparison between PQRST, LVEF, and traditional predictive models by ROC curve analysis to predict 24-month all-cause mortality.

	**Difference between area (95% CI)**	***P*-value**
PQRST vs. LVEF	0.050 (-0.051–0.150)	0.331
PQRST vs. GRACE	0.126 (0.048–0.203)	0.002
PQRST vs. TIMI	0.011 (-0.106–0.129)	0.850
GRACE vs. TIMI	0.114 (-0.001–0.230)	0.052
GRACE vs. LVEF	0.175 (0.088–0.263)	<0.001
TIMI vs. LVEF	0.061 (-0.655–0.188)	0.345

*Abbreviations as in Table 1*.

### Association of PQRST Score With Increased 24-Month All-Cause Mortality, Similar to LVEF, GRACE and TIMI Scores

**Figure 3:**
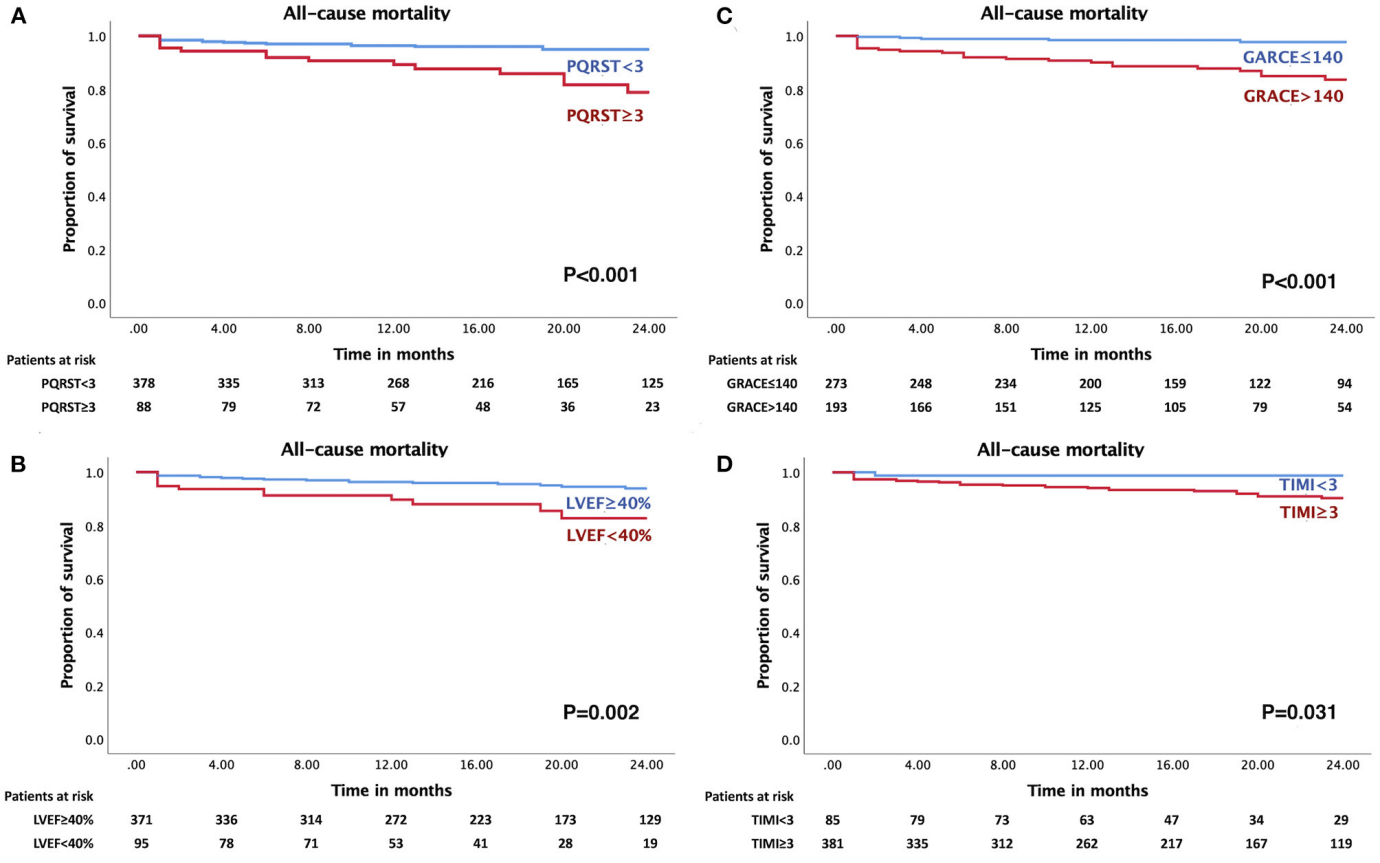
Cumulative survival probabilities of all-cause mortality stratified by PQRST, LVEF, and traditional predictive models. Increased 24-month all-cause mortality rate in NSTE-ACS patients with **(A)** PQRST score ≥ 3 (*p* < 0.001), **(B)** LVEF < 40% (*p* = 0.002), **(C)** GRACE score > 140 (*p* < 0.001), and **(D)** TIMI score ≥ 3 (*p* = 0.031).

Of the 466 patients in this study, 30 all-cause mortality events occurred during the 24-month observation period. Subjects with events had a higher PQRST score of 2.42 ± 1.76 than subjects without events (1.27 ± 1.53) (*p* < 0.001). Kaplan-Meier analysis showed that 15.9% (14/88) of patients with PQRST ≥ 3 died at 24 months compared to 4.2% (16/378) of patients with PQRST < 3 (*p* < 0.001) (Figure 3A). Moreover, there was a significant increase in mortality in patients with LVEF < 40% (12/95, 12.6%) compared to LVEF ≥ 40% (18/371, 4.9%) (*p* = 0.002) (Figure 3B); with GRACE > 140 (25/193, 13%) compared to GRACE ≤ 140 (5/273, 1.8%) (*p* < 0.001) (Figure 3C); and with TIMI ≥ 3 (29/381, 7.6%) compared to TIMI < 3 (1/85, 1.2%) (*p* = 0.031) (Figure 3D).

### Twenty-Four-Month All-Cause Mortality Stratified by PQRST Score ≥ 3 in Subgroups With Varying LVEF, GRACE and TIMI Scores

**Figure 4:**
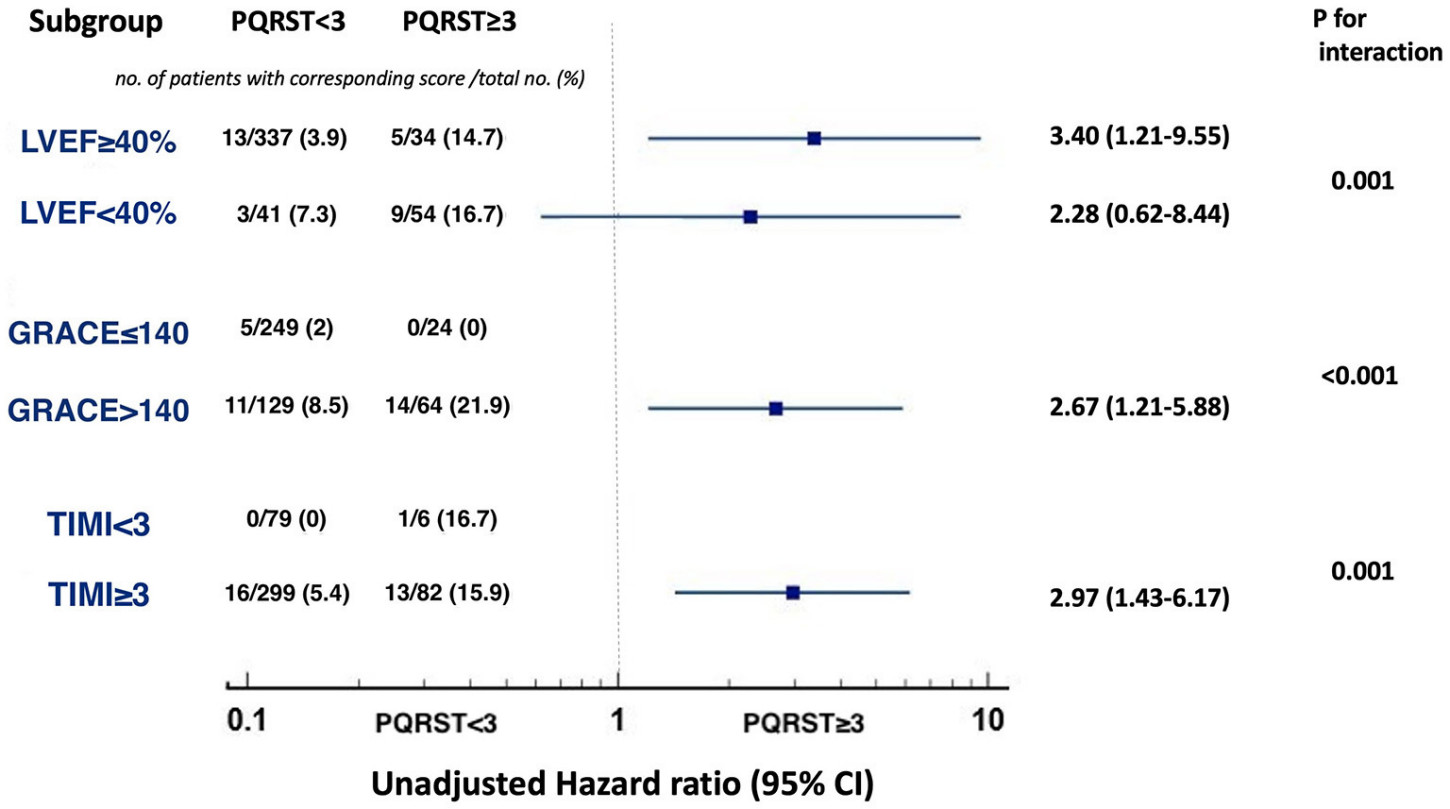
Predictive value of PQRST score for 24-month all-cause mortality in the subgroups with varying LVEF, GRACE, and TIMI scores. A PQRST score ≥ 3 increased risk of 24-month all-cause mortality in the subgroups of LVEF ≥ 40% (*p* = 0.020), GRACE score > 140 (*p* = 0.015), TIMI score ≥ 3 (*p* = 0.004). No significant difference was attained in the subgroup of LVEF < 40% (*p* = 0.216). No event occurred in the patients with GRACE ≤ 140 and PQRST score ≥ 3, and with TIMI < 3 and PQRST score < 3.

Figure 4 showed the predictive value of PQRST score for 24-month all-cause mortality in various subgroups of interest. In the subgroups of patients with LVEF ≥ 40%, GRACE > 140, and TIMI ≥ 3, PQRST ≥ 3 could still stratify populations at increased risk of 24-month mortality compared to those with PQRST < 3 (unadjusted hazard ratio [HR]: 3.40; 95% CI: 1.21 to 9.55; *p* = 0.020 in LVEF ≥ 40%; HR: 2.67; 95% CI: 1.21 to 5.88; *p* = 0.015 in GRACE > 140; HR: 2.97; 95% CI: 1.43 to 6.17; *p* = 0.004 in TIMI ≥ 3) (*p* values for interaction, respectively: 0.001, < 0.001, and 0.001) (Figure 4). However, 24-month mortality was not significantly associated with PQRST ≥ 3 compared with PQRST < 3 in the subgroup of LVEF < 40% (HR: 2.28; 95% CI: 0.62–8.44; *p* = 0.216). No event occurred in the patients with GRACE ≤ 140 and PQRST score ≥ 3 (0/24), and with TIMI < 3 and PQRST score < 3 (0/79).

## discussion

In the present study, we identified 4 out of 9 ECG patterns associated with LVEF reduction to < 40% in an NSTE-ACS population (derivation cohort, *n* = 353). With the first ECG at the triage of the emergency room, we developed the PQRST score, which can successfully predict LVEF < 40%, with an AUC value of 0.824; the cutoff point was 3 at a sensitivity of 53.7% and a specificity of 90.2%. This major finding was also tested in a validation cohort (*n* = 113) with an AUC value of 0.899.

### Risk Stratification in NSTE-ACS

The versatile clinical manifestations of NSTE-ACS have a wide spectrum from stable coronary artery disease, stable heart failure to event of equivalent emergency of ST-elevation myocardial infarction (STEMI) or even to critical illness with life-threatening hemodynamic instability. Instead of emphasizing achieving door-to-ECG and door-to-balloon time in STEMI scenarios (30, 31), prompt detection of LV systolic dysfunction is more crucial in risk stratification in NSTE-ACS. ECG patterns have been previously reported as screening tools to identify LV systolic dysfunction in various populations. To detect severe LV systolic dysfunction (LVEF < 35%), Chugh and colleagues (15) analyzed the ECG tracings of 1,047 patients from the community-based Oregon Sudden Unexpected Death Study. Six ECG patterns independently associated with LVEF < 35% (QRS duration > 110 ms, QRS transition > V4, HR > 85 bpm, delayed intrinsicoid deflection ≥ 50 ms, frontal QRS-T angle > 90% and QTc interval ≥ 460 ms) and approximately 33% of patients with severe LV systolic dysfunction were identified if there were more than four abnormal ECG markers. To unveil the underdiagnosis and undertreatment of LV systolic dysfunction (LVEF < 40%) in the elderly, Olesen and colleagues (17) analyzed 260 ECG tracings and reported that 90% LV systolic dysfunction may be detected by identifying elevated NT-proBNP > 35 pmol/L, Q wave, atrial fibrillation and pacing/LBBB/ORS duration > 120 ms. Since ECG is the examination with the highest timeliness, good availability, and robust reliability, we designed the present study to test this concept in the NSTE-ACS scenario. Our predictive model included similar ECG patterns, such as QRS duration > 110 ms, HR > 100 bpm, and PRWP. In addition, according to the logistic regression, we also gave different weights to each ECG pattern to predict LV systolic dysfunction. In contrast to the aforementioned studies, the distribution of ST depression was included in our predictive model because its extent was highly associated with the severity of myocardial ischemia and poor prognosis in acute coronary syndrome (19, 32). The PQRST score seemed to have less prognostic value than the GRACE score in all-cause mortality. However, LV systolic function was not included in either the GRACE or TIMI score, and Syyli et al. (33) reported the additive prognostic value of LVEF beyond the GRACE score to predict 6-month all-cause mortality. In the present study, the PQRST score not only has similar additive prognostic value on these traditional scoring systems but also has additive prognostic value for those with LVEF ≥ 40%. This might imply that this ECG-derived PQRST score can reflect electrical, structural, and functional changes in the myocardium. As a result, the PQRST score can help clinicians be aware of possible left ventricular systolic dysfunction immediately at triage.

### Decision on Pretreatment With P2Y12 Receptor Antagonist

Approximately 5–10% of patients with NSTE-ACS require CABG (34) and have higher perioperative bleeding complications if they are under dual antiplatelet therapy (35, 36). The updated ESC guidelines in 2020 argue against routine pretreatment with P2Y_12_ receptor inhibitors in patients whose coronary anatomy is not known (18). One of the considerations is increasing perioperative bleeding risk or delaying CABG timing when patients routinely take P2Y_12_ receptor inhibitors. In our cohort, patients with LVEF < 40% were more likely to undergo CABG (34.7%, *p* < 0.001). The advantage of early LV systolic function assessment is that it can identify patients who may need further CABG and avoid the loading dose of P2Y_12_ receptor inhibitors.

### Point of Care Ultrasound in NSTE-ACS

POCUS is increasingly applied in medical emergencies and critical care (11). As mentioned above, timely LV systolic function assessment is important in NSTE-ACS patients. However, cardiac POCUS is still not well democratized in such scenarios. Although a previous study reported that patients were more likely to be discharged on guideline-directed medical therapies if in-hospital LV systolic function assessments were performed (9), the optimal timing of performing echocardiography has not yet been reported. In patients with regional wall motion abnormalities (RWMA), the reporting of LVEF can be even more difficult for non-cardiologists or cardiologists without specific expertise. Fifty Nine Percentage of these NSTE-ACS patients underwent cardiac POCUS examinations in the present study, which were ordered by cardiology consultants. In summary, the PQRST score ≥ 3 at emergency triage might serve as a red flag to initiate quick consultation for early cardiac POCUS and remind cardiologists to pay more attention to possible RWMA and to avoid overestimation of LVEF.

### Artificial Intelligence-Assisted Pipeline in NSTE-ACS

Artificial intelligence (AI) has successfully dealt with subjects assessing image quality (37) and recognizing regional wall motion abnormalities on echocardiography (38). The present study is also a small-scale annotation step for developing a possible AI model to identify LVEF < 40% based on standard 12-lead ECG. Recently, AI-enabled tools have also been reported to guide critical care physicians with no formal training in ultrasound to obtain cardiac POCUS images (39). All these techniques provide a potential AI-assisted pipeline to perform delicate and prompt risk stratification for NSTE-ACS.

### Study Limitation

There were several limitations in our study. First, this was a retrospective study that included a small population in a single center. However, this is because we only included patients whose echocardiographic studies were completed within 72 h after the first triage ECG acquisition, and there were no coronary interventions in between. Second, the PQRST score was only tested in the NSTE-ACS population. Whether the predictive value of the PQRST score could be generalized to other clinical scenarios, such as non-ischemic cardiomyopathy and valvular heart disease, warranted further large-scale prospective studies. Third, the outcome prediction ability of the PQRST score was inferior to that of the GRACE score; nevertheless, the prognostic ability of the PQRST score was like that of LVEF and TIMI score in all-cause mortality. Furthermore, the PQRST score could provide incremental prognostic value for populations with GRACE score > 140 and TIMI score ≥ 3 regarding the study endpoint. After all, GRACE and TIMI scores could not be completed in the emergency room triage and should depend on history taking, blood biomarkers and hemodynamic data. As a result, the PQRST score could provide earlier risk stratification.

## Data Availability Statement

The raw data supporting the conclusions of this article will be made available by the authors, without undue reservation.

## Ethics Statement

The studies involving human participants were reviewed and approved by CHGH-IRB No: (856)110-02. IRB in Cheng Hsin General Hospital. The ethics committee waived the requirement of written informed consent for participation.

## Author Contributions

W-CL: drafting and revising the manuscript, analysis of data, and collection and interpretation of data. M-CH: instructor of cardiac POCUS examination, echocardiography image analysis, and revising the manuscript. W-HY: founder for database of chest pain center at Cheng Hsin General Hospital and revising the manuscript. T-PT and W-TL: collection and interpretation of data and revising the manuscript. K-CH: conception and design, analysis of data, and revising the manuscript. All authors contributed to the article and approved the submitted version.

## Conflict of Interest

The authors declare that the research was conducted in the absence of any commercial or financial relationships that could be construed as a potential conflict of interest.

## Publisher's Note

All claims expressed in this article are solely those of the authors and do not necessarily represent those of their affiliated organizations, or those of the publisher, the editors and the reviewers. Any product that may be evaluated in this article, or claim that may be made by its manufacturer, is not guaranteed or endorsed by the publisher.

## Abbreviations

ECG: electrocardiography
NSTE-ACS: non-ST segment elevation acute coronary syndrome
STD: ST-segment depression
PRWP: poor R wave progression
LVEF: left ventricular ejection fraction
POCUS: point of care ultrasounds
TIMI: Thrombolysis In Myocardial Infarction
GRACE: Global Registry of Acute Coronary Events
RWMA: regional wall motion abnormalities.
